# Evaluating denoising strategies in resting‐state functional magnetic resonance in traumatic brain injury (EpiBioS4Rx)

**DOI:** 10.1002/hbm.25979

**Published:** 2022-06-20

**Authors:** Marina Weiler, Raphael F. Casseb, Brunno M. de Campos, Julia S. Crone, Evan S. Lutkenhoff, Paul M. Vespa, Martin M. Monti, for the EpiBioS4Rx Study Group

**Affiliations:** ^1^ Department of Psychology University of California Los Angeles Los Angeles California USA; ^2^ Neuroimaging Laboratory University of Campinas Campinas São Paulo Brazil; ^3^ David Geffen School of Medicine University of California Los Angeles Los Angeles California USA; ^4^ Department of Neurosurgery, Brain Injury Research Center University of California Los Angeles Los Angeles California USA

**Keywords:** artifact, head motion, motion correction, nuisance regression, physiological noise, TBI

## Abstract

Resting‐state functional MRI is increasingly used in the clinical setting and is now included in some diagnostic guidelines for severe brain injury patients. However, to ensure high‐quality data, one should mitigate fMRI‐related noise typical of this population. Therefore, we aimed to evaluate the ability of different preprocessing strategies to mitigate noise‐related signal (i.e., in‐scanner movement and physiological noise) in functional connectivity (FC) of traumatic brain injury (TBI) patients. We applied nine commonly used denoising strategies, combined into 17 pipelines, to 88 TBI patients from the Epilepsy Bioinformatics Study for Anti‐epileptogenic Therapy clinical trial. Pipelines were evaluated by three quality control (QC) metrics across three exclusion regimes based on the participant's head movement profile. While no pipeline eliminated noise effects on FC, some pipelines exhibited relatively high effectiveness depending on the exclusion regime. Once high‐motion participants were excluded, the choice of denoising pipeline becomes secondary ‐ although this strategy leads to substantial data loss. Pipelines combining spike regression with physiological regressors were the best performers, whereas pipelines that used automated data‐driven methods performed comparatively worse. In this study, we report the first large‐scale evaluation of denoising pipelines aimed at reducing noise‐related FC in a clinical population known to be highly susceptible to in‐scanner motion and significant anatomical abnormalities. If resting‐state functional magnetic resonance is to be a successful clinical technique, it is crucial that procedures mitigating the effect of noise be systematically evaluated in the most challenging populations, such as TBI datasets.

## Graphical abstract

In this study, we evaluated denoising strategies in traumatic brain injury patients from the EpiBioS4Rx trial. While no pipeline eliminated noise effects on functional connectivity, some pipelines exhibited relatively high effectiveness depending on the exclusion regime; pipelines combining spike regression with physiological regressors were the best performers. We provide a framework and guidelines for fMRI research in traumatic brain injury.

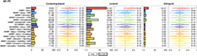

## 1 INTRODUCTION

Over the last few decades, the assessment of spontaneous oscillations in the blood oxygenation level‐dependent (BOLD) measured by resting‐state functional magnetic resonance (rsfMRI) has increasingly been used to aid diagnosis and prognosis in neurological disorders (Baker et al., 2014; de Vos et al., 2018; Franzmeier et al., 2020; Wolters et al., 2019; Woodward et al., 2012). Yet, despite the appeal and wide adoption of this technique, it suffers from significant limitations for distinguishing oscillations associated with neural activity from those induced by nonneural sources (Birn, 2012; Murphy et al., 2013; Power et al., 2017). In‐scanner head motion can systematically generate artifactual correlations across brain regions and spurious functional connectivity (FC) results regardless of how they are assessed (e.g., seed‐based analysis, graph theory, and amplitude of low frequency fluctuations) (Power et al., 2012; Satterthwaite et al., 2012; Van Dijk et al., 2012).

In the context of severe brain injury and disorders of consciousness, some international guidelines (Kondziella et al., 2020) now suggest incorporating rsfMRI in the diagnostic process given its ability to complement bedside neurobehavioral assessments and provide prognostic information (Demertzi et al., 2019; Madhavan et al., 2019; Silva et al., 2015; Vanhaudenhuyse et al., 2010). Controlling for head motion in clinical samples, however, represents a major obstacle when compared to healthy participants given the patients' tendency to be more restless and noncooperative and, in turn, exhibit more movement in the scanner. Patients with traumatic brain injury (TBI), specifically, present frequent abnormal movements (alternatively described as dyskinesias or “paroxysmal” motor phenomena) such as posturing, shivering, and seizures, which dominate the early period following TBI, while other abnormal movements, including tremor, dystonia, tics, parkinsonism, and chorea, may be seen several months following TBI (Hannawi et al., 2016).

While this issue could be mitigated with the use of sedative agents, these will affect any subsequent analysis of brain network function (Monti et al., 2013), thus making the development of analytical approaches to mitigating in‐scanner motion a more desirable strategy. In this sense, a large number of analysis pipelines have been proposed to address the issue (Muschelli et al., 2014; Power et al., 2015). However, most of this work has been developed and evaluated in neurotypical individuals or clinical populations that do not usually present significant anatomical and functional abnormalities (Burgess et al., 2016; Ciric et al., 2018; Parkes et al., 2018; Power et al., 2020; Raval et al., 2020). No pipeline has ever been validated with respect to patients exhibiting the degree of in‐scanner motion (Hannawi et al., 2016; Monti et al., 2015) and the extensive brain pathology (such as atrophy and trauma‐induced deformations) known to lead to suboptimal and biased performance of conventional analysis software (Lutkenhoff et al., 2014). If rsfMRI is to be a successful technique used in routine clinical practice (Kondziella et al., 2020), it is crucial that procedures mitigating the effect of noise be systematically evaluated also in the most challenging populations.

To address this gap, we extend a prior large‐scale evaluation of different pipelines (Parkes et al., 2018) to the very challenging population of moderate‐to‐severe TBI to provide a quantitative comparative assessment of different denoising strategies. Specifically, we applied nine commonly used denoising strategies, combined into 17 pipelines, to TBI patients from the Epilepsy Bioinformatics Study for Anti‐epileptogenic Therapy clinical trial (EpiBioS4Rx) (Vespa et al., 2019) and evaluated the ability of each one to remove noise from the BOLD signal. We conclude by providing a framework for clinicians and translational scientists interested in using fMRI to select the pipeline that balances the ability to mitigate noise with the constraints and aims of their study.

## 2 METHODS

### 2.1 Subjects

This study included 88 patients from the EpiBioS4Rx dataset, a longitudinal study that aims to discover and validate observational biomarkers of epileptogenesis after TBI (Vespa et al., 2019). As described elsewhere, patients were enrolled across 12 sites within 72 h following TBI involving frontal and/or temporal hemorrhagic contusion, according to criteria previously published (Vespa et al., 2019). Our sample consisted of 21 females and 67 males, with mean age of 41.1 (7–84) years, level of consciousness after TBI measured by the Glasgow Coma Scale at the emergency department arrival (Teasdale & Jennett, 1974) of 7.8 (1–15), and time since injury of 11 (0–36) days. Informed consent was obtained from a surrogate family member or legally authorized representative, using IRB‐approved consent methods.

### 2.2 Image acquisition and processing

Data were acquired on 1.5 or 3 T MR system, including an anatomical (T1‐weighted) and functional (T2*‐weighted echo‐planar images) acquisitions (see Tables S1 and S2 for detailed parameter listing). Data were processed using code adapted from Parkes et al. (2018) (https://github.com/lindenmp/rs-fMRI).

The T1‐weighted high‐resolution structural image was processed using the following steps: neck and lower head removal using FSL's *robustfov*, and segmentation into white matter (WM), cerebrospinal fluid (CSF), and grey matter (GM) probability maps using SPM8's New Segment. To minimize WM/CSF signal correlation with the GM mask, we applied five erosion cycles to the WM mask and two erosion cycles to the CSF mask following extraction of the ventricles (i.e., masking out of the CSF surrounding the sulci/gyri of the cortex; Power et al., 2017), followed by a nonlinear spatial transform to MNI space using Advanced Normalization Tools (ANTs; Avants et al., 2008) with default settings (using the *antsRegistrationSyN.sh* script).

All functional data underwent a common set of preprocessing steps, before and after the denoising step, which consisted of: remotion of the first four volumes using FSL's *fslroi*; realignment of volumes to acquire raw motion parameters, slice‐time correction, and realignment of all volumes to the first volume (first pass) and then to the mean volume (second pass) using SPM8; co‐registration of EPI data to the native, cropped, T1‐weighted image via rigid‐body registration using ANTs; application of the inverse nonlinear transform derived from the T1‐weighted image processing pipeline to the co‐registered EPI data using ANTs; linear detrending of the spatially normalized BOLD time series; intensity normalization of the EPI data to mode 1000 units and finally, application of the denoising pipeline. After each registration step, we visually confirmed accurate alignment in all three dimensions using FSLView. Following the denoising, images were filtered with a band‐pass 0.008–0.08 Hz using REST v1.8, and spatially smoothed with a 6 mm FWHM kernel using SPM8 (with an exception for ICA‐AROMA, which requires smoothing before noise correction).

### 2.3 Denoising strategies

Denoising is achieved by removing variance attributable to head motion and respiration/cardiac‐induced noise from the BOLD signal. What is debated is how to best measure, operationalize, and remove these sources of noise. Table 1 summarizes the denoising approaches used in our analysis. We combined these approaches into 17 pipelines, as done in prior work that used a different clinical sample (Parkes et al., 2018).

**TABLE 1:** Denoising strategies

Head displacement	Head motion parameters (HMPs): Six parameters (three rotations and three translations about/along the x‐, y‐, and z‐axes) included as noise regressors (6HMP). Additional regressors derived from the six parameters (e.g., temporal and quadratic terms of each parameter, as well as their difference) are often included to account for delayed and nonlinear motion‐induced spin history effects (24HMP; Friston et al., 1996).
	Spike regression (Satterthwaite et al., 2013): For each volume containing excessive motion, a separate regressor is generated containing a value of 1 at that volume, and 0 at all others. Volumes are considered contaminated if FD_Jenk_ >0.25 mm. FD_Jenk_ represents the root mean squared of the six motion parameters (Jenkinson et al., 2002).
	Scrubbing (Power et al., 2014): Each volume containing excessive motion is removed from the time series if FD_Power_ >0.2 mm or DVARS >2%. After removal, uncontaminated segments of BOLD data lasting fewer than five contiguous volumes are also removed. FD_Power_ represents the sum of the absolute values of the differentiated realignment estimates (by backward differences) at every time point.
Physiology‐related	Physiological regressors (2phys): Regression of the average signal from WM and CSF, tissues not expected to exhibit BOLD oscillations tied to neural activity.
	Anatomical Component‐Based Correction (aCompCor; Behzadi et al., 2007/aCompCor50; Muschelli et al., 2014): This approach involves extracting orthogonal components of temporal variance from voxel‐wise time series for the WM and CSF masks separately. Then, either the five components with greater eigenvalue for each tissue are included in the denoising regression (aCompCor), or as many components as needed to cumulatively explain at least 50% of the variance in each tissue (aCompCor50).
Mixed approaches	Global signal regression (GSR): Regression of the average signal across all the voxels of the brain.
	ICA‐based Automatic Removal Of Motion Artifacts (ICA‐AROMA; Pruim, Mennes, van Rooij, et al., 2015): Automated data‐driven method to identify and remove via regression motion‐related independent components.

Abbreviations: BOLD, blood oxygenation level‐dependent; CSF, cerebrospinal fluid; DVARS, derivative of the root mean squares variance over voxels; FD, framewise displacement; ICA, independent component analysis; WM, white matter.

### 2.4 Head movement estimation (in‐scanner motion)

As shown in Table 1, some pipelines rely on the ability to pinpoint volumes corrupted by excessive motion. In general, motion in a volume is quantified by the Derivative of the root mean squares VARiance over voxelS (DVARS; Power et al., 2012; Smyser et al., 2011) and framewise displacement (FD; Jenkinson et al., 2002; Power et al., 2012). In this study and consistent with previous work (Parkes et al., 2018), we used the Jenkinson method (FD_Jenk_; Jenkinson et al., 2002) to determine FD for spike regression and for the exclusion criteria, but the Power method (Power et al., 2012) to determine FD for scrubbing.

### 2.5 Participant exclusion regimes

Finally, it is debated how to determine the threshold at which a subject contains excessive motion and thus should be discarded from any analysis. We compared the performance of all pipelines under three different participant exclusion regimes: (i) censoring‐based, (ii) lenient, and (iii) stringent (Table 2 shows the criteria for subject exclusion in each regime). The criterion for the censoring‐based regime was data less than 4 min. Using the mean FD_Jenk_ (Jenkinson et al., 2002) across all volumes (hereafter, mFD), the exclusion criterion for the lenient regime was mFD >0.55 mm. For the stringent regime, the exclusion criteria were if any of the following criteria were applicable: (i) mFD >0.25 mm; (ii) more than 20% of the FDs were above 0.2 mm; and (iii) if any FDs were greater than 5 mm.

**TABLE 2:** Participant exclusion regimes and their criteria for exclusion

Regime	Exclusion criteria
Censoring‐based (Satterthwaite et al., 2013; Van Dijk et al., 2012)	Excluded subjects when less than 4 min of noncontaminated volumes remained after volume censoring (<4 min of data).
Lenient (Satterthwaite et al., 2012)	Excluded subjects if:
	(i) < 4 min of data; or
	(ii) high levels of head gross motion, defined as mFD >0.55 mm.
Stringent (Satterthwaite et al., 2013)	Excluded subjects if:
	(i) < 4 min of data;
	(ii) mFD >0.25 mm;
	(iii) more than 20% of the volumes presented FD_Jenk_ >0.2 mm; or
	(iv) any volume presented FD_Jenk_ >5 mm.

Abbreviations: FD, framewise displacement; mFD, mean framewise displacement.

### 2.6 Quantification and comparison of the exclusion regimes

To compare mFD across different exclusion regimes, we implemented a Kruskal‐Wallis test with a Bonferroni correction for multiple comparisons. To ensure that neither the diagnoses nor time since injury related to in‐scanner head motion, we implemented a Pearson's correlation (*r*) with mFD and the total Glasgow Coma Scale score or time since injury, respectively. In addition, we implemented a logistic regression to determine the effects of confound variables such as age, gender, time since injury, and Glasgow Coma Scale on the likelihood that participants would fall into the Stringent regime. All statistical analysis was performed using SPSS Statistics for Windows, version 27.0 (SPSS Inc.).

### 2.7 QC measures

After image preprocessing, we used a template containing 333 cortical regions (ROIs; Gordon et al., 2016) to define the areas to extract gray matter (GM)‐weighed denoised time series for further analysis. We then calculated FC as Pearson's correlation coefficient (*r*) between each pair of ROI time series, then implemented a Fisher's *r*‐to‐*z* transformation. The FC matrices obtained following each denoising pipeline were then used to evaluate the ability of each pipeline to remove noise‐induced correlations by the two QC measures described below.

#### 2.7.1 QC‐FC correlation

Represents the correlation between FC and in‐scanner head motion (mFD) since nonneuronal fluctuations can increase the apparent FC between regions by introducing spurious common variance across time series. Here, we calculated Pearson's correlation coefficient (*r*) using MATLAB's *corr* function (MATLAB 2020a, The MathWorks, Inc.) between each pair of ROIs FC and the mFD across patients. Then, we compared the proportion of edges where this QC‐FC correlation was statistically significant, as well as the median absolute QC‐FC correlation after applying each denoising pipeline. Higher QC‐FC (either the proportion of significant correlations or the absolute *r*‐value) represents the inability of a pipeline to mitigate noise in FC.

#### 2.7.2 QC‐FC distance‐dependence

Indicates whether the correlation between FC and in‐scanner head motion (mFD) is spatially structured—a known feature of motion‐induced artifacts (Power et al., 2012, 2014; Satterthwaite et al., 2012; Van Dijk et al., 2012).

It should be noted that spatial smoothing also plays a role in distance‐dependent FC measurements, inducing spurious correlation due to the signal “smearing” to neighboring voxels, especially with higher Gaussian smoothing kernels (Alakörkkö et al., 2017). While the optimal filter size for analysis of fMRI depends on various criteria and specific functional areas and experimental tasks, we opted to use a 6 mm FWHM kernel size, which corresponds to roughly two times the size of our voxel, as per recommendation (Mikl et al., 2008; Pajula & Tohka, 2014), and previous reports (Parkes et al., 2018). Our choice of a relatively small kernel might help control for the undesirable and intrinsic smoothing‐related effects on distance‐dependent FC measurements.

Here, we calculated the distance between ROIs as the Euclidean distance (MATLAB's *pdist2* function) between the stereotaxic coordinates of the volumetric centers of ROI pairs. We quantified the relationship between this distance and the QC‐FC correlation for each edge using Spearman's rank correlation coefficient (*ρ*) due to the nonlinearity of some associations using MATLAB's *corr* function. Higher QC‐FC distance‐dependence values represent the inability of a pipeline to mitigate spatially structured noise in FC.

#### 2.7.3 Loss of temporal degrees of freedom (tDOF‐loss)

The third QC benchmark measured the ability of each pipeline to retain statistical power during the denoising process (tDOF), which varies according to the original time series length and the number of regressors used in the model. Given a fixed number of regressors, the tDOF will be higher with longer time series, and vice‐versa. In the present work, all datasets contained 300 time points, allowing us to control as much as we could to keep tDOF constant across datasets.

The number of regressors used in a model, however, is not constant across pipelines (see Table S3), because they represent the number of time points and/or the number of regressors used to denoise the data. Generally speaking, high‐motion subjects present more regressors because they either present more contaminated time points in case of volume censoring, or more components classified as noise in case of data‐driven strategies. Thus, the performance of denoising strategies must be balanced against lost temporal degrees of freedom (tDOF‐loss).

## 3 RESULTS

### 3.1 Participant exclusion

**FIGURE 1:**
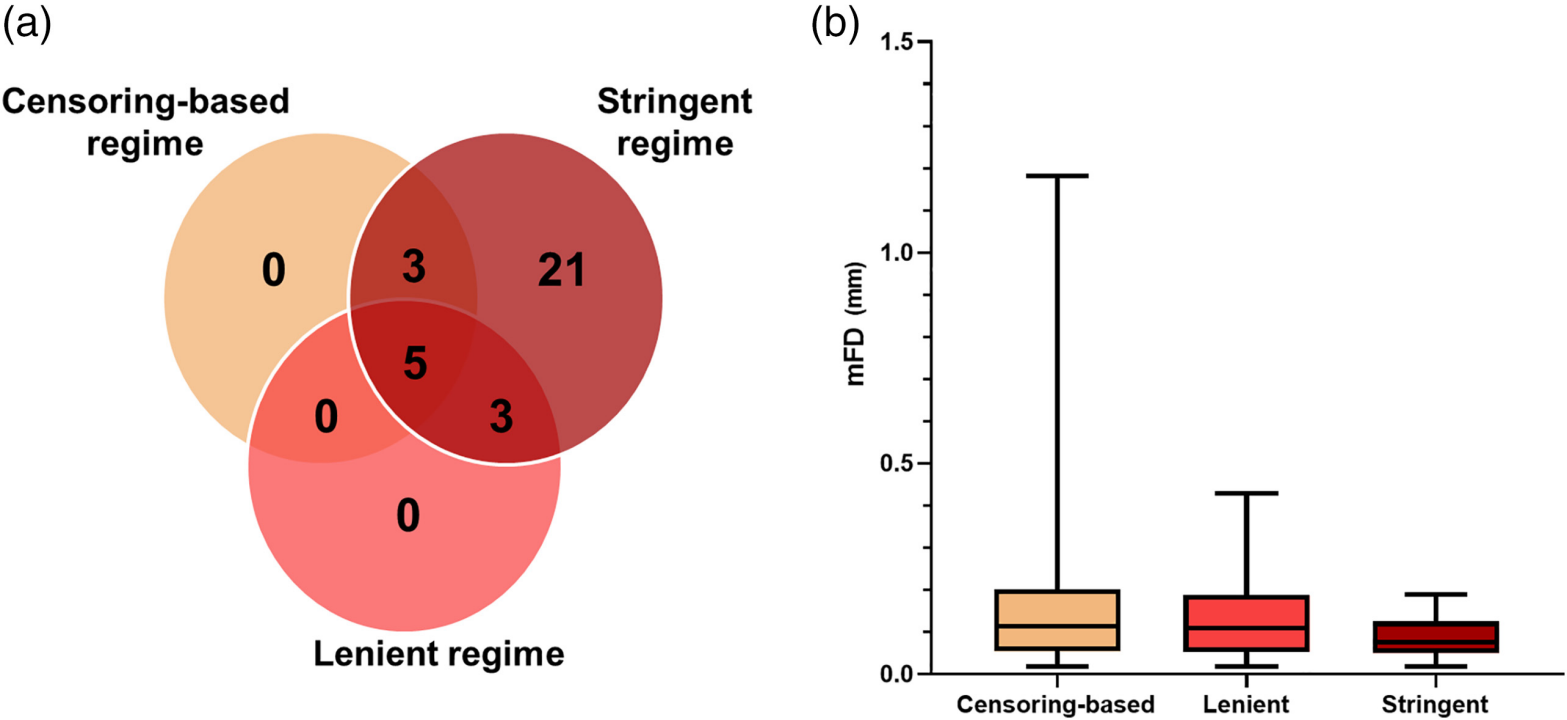
(a) Number of participants excluded in each regime; (b) box plots of the mFD values for each regime. mFD, mean framewise displacement.

As shown in Figure 1a, censoring‐based, lenient, and stringent regimes resulted in the exclusion of 8, 11, and 32 patients (9%, 12.5%, and 36%, respectively). As expected, participants under the stringent regime presented significantly smaller mFD compared to censoring‐based and lenient regimes (Figure 1b; Kruskal–Wallis test, *H[2]* = 9.791, *p* = .0075; mean rank mFD 116.45 for censoring‐based, 113.18 for lenient, and 85.00 for stringent). Pairwise comparisons Bonferroni‐adjusted for multiplicity: stringent versus lenient, *p* = .028; stringent versus censoring‐based, *p* = .01; lenient versus censoring‐based, *p* = 1. In‐scanner head movement did not correlate with Glasgow Coma Scale (Pearson *r[84]* = 0.080, *p* = .462), age (Pearson *r[85]* = 0.139, *p* = .201), nor time since injury (Pearson *r[79]* = 0.128, *p* = .253). The logistic regression model, implemented to exclude the effects of confounds on the likelihood that patients would fall into the stringent regime, was not statistically significant, *χ*2(4) = 6.051, *p* = .195. The model explained 10% (Nagelkerke *R* ^*2*^) of the variance in Stringent regime and correctly classified 63% of cases.

### 3.2 QC measures

**FIGURE 2:**
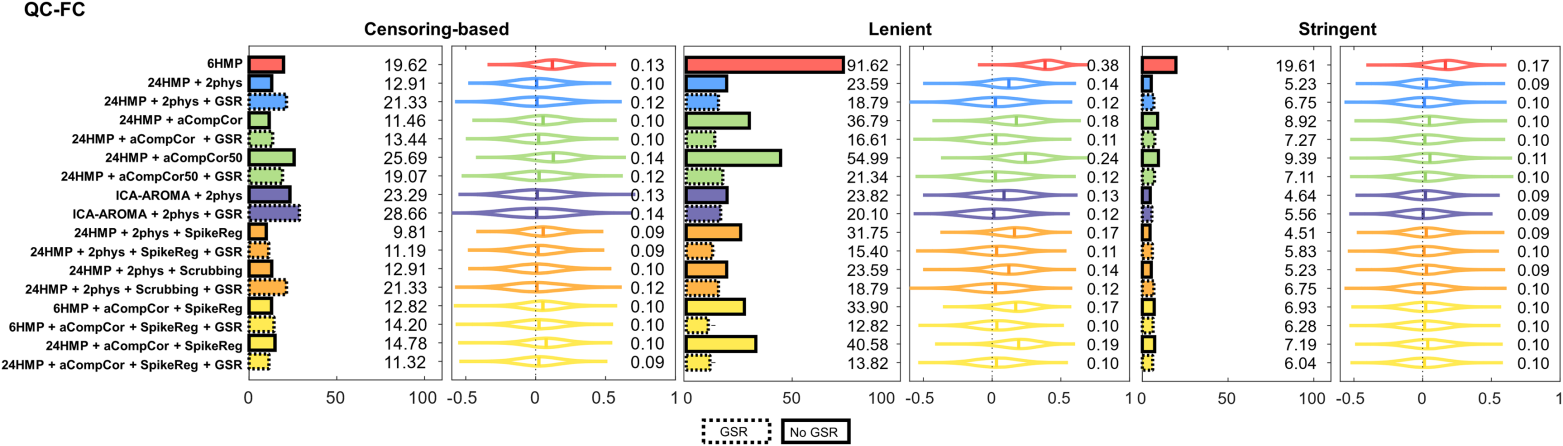
QC‐FC correlations under the three regimes of participant exclusion. On the left of each panel, results are shown as the proportion of significant FCs that correlated with the patient's head movement (mFD), *p* < .05. On the right of each panel, results are shown as the full distribution of QC‐FC, and the corresponding median value. Better denoising pipelines result in fewer correlations between FC and head movement, giving values closer to 0. FC, functional connectivity; GSR, global signal regression; mFD, mean framewise displacement; QC, quality control.

**FIGURE 3:**
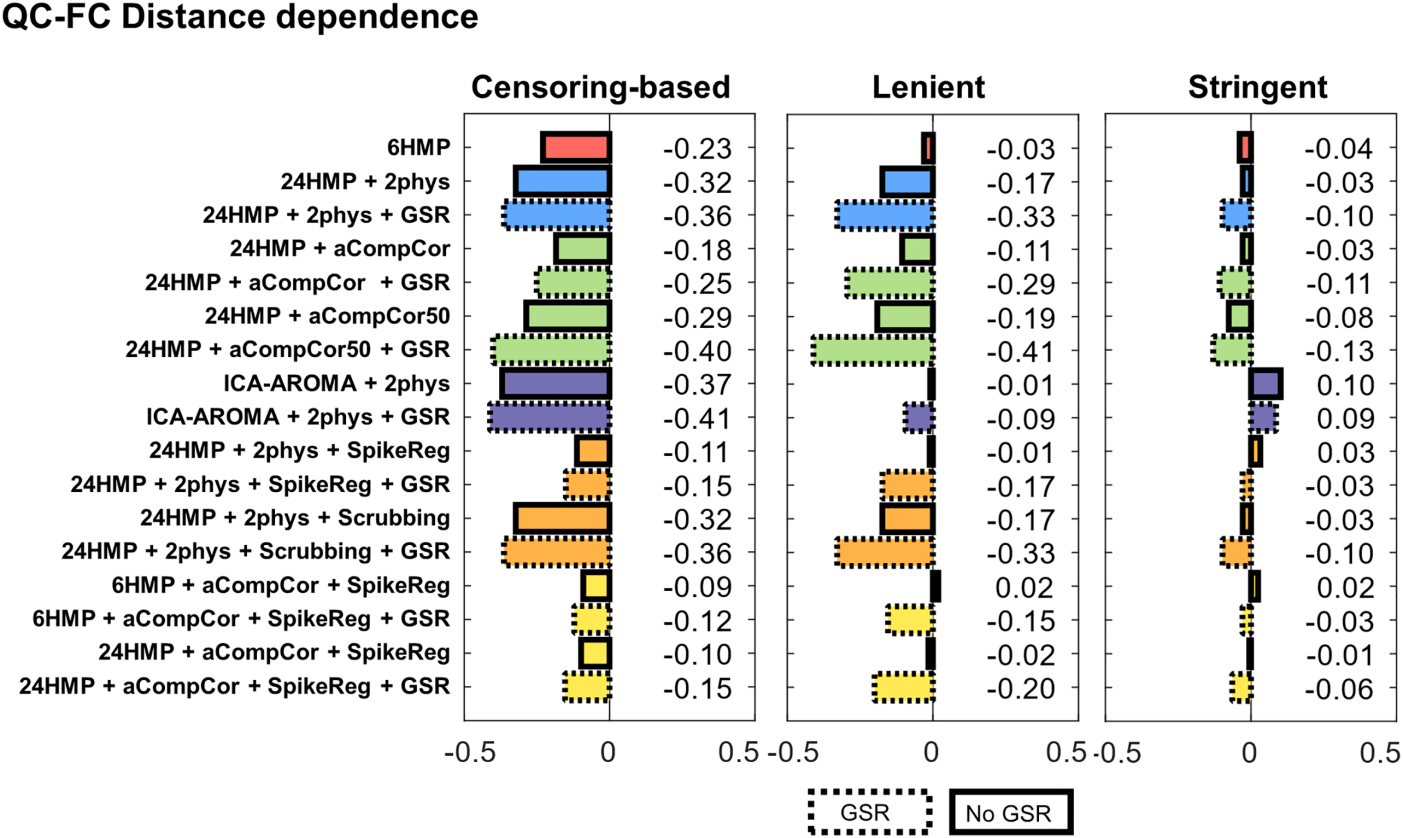
QC‐FC distance‐dependence under the three participant exclusion regimes. Results are presented as Spearman's *ρ* correlation coefficient. Better denoising pipelines result in fewer correlations between FC and head movement, giving values closer to 0. FC, functional connectivity; GSR, global signal regression; QC, quality control.

As shown in Figures 2 and 3, consistent with prior work (Parkes et al., 2018), while no pipeline entirely eliminated noise‐related effects on FC patterns, some pipelines exhibited relatively high effectiveness at mitigating it depending on the exclusion regime.

#### 3.2.1 QC‐FC

Overall, no pipeline in any exclusion regime reduced the effect of noise to zero (Figure 2). Nonetheless, our results show that some pipelines, under a given regime, perform better than others.

First, the censoring‐based and lenient regimes resulted in approximately 10%–29% and 13%–55% proportion of significant correlations and absolute *r*‐values between 0.09–0.14 and 0.10–0.24, respectively. 6HMP pipeline was an outlier with ~92% proportion of QC‐FC significant correlations and an absolute correlation between motion and FC of 0.38 in the lenient regime. In comparison, the stringent criterion resulted in lower QF‐FC across all pipelines, reducing the correlations significantly to less than 10% and median *r*‐value between motion and FC to 0.09–0.11 (with the sole exception of the 6HMP, with ~20% significant correlations).

Second, within each of the three exclusion regimes, different strategies exhibit different effectiveness at mitigating noise. Overall, in the (i) censoring‐based regime, the best performance was obtained with different combinations of 24HMP, aCompCor, and spike regression. Conversely, the three worst‐performing pipelines all featured data‐driven methods, including aCompCor50 and ICA‐AROMA. The addition of GSR generally resulted in the worsening of pipeline performance. Under the (ii) lenient regime, a very different pattern of results was observed. Overall, the best performing pipelines under this regime were the two featuring aCompCor, spike regression, and GSR, with either 6 or 24 HMP. At the opposite end of performance, pipelines without GSR underperformed those with GSR, and the pipeline with 6HMP alone resulted in the slightest mitigation of noise‐induced effects on FC. The inclusion of GSR improved pipeline performance for all pipelines, with the greatest benefit observed for the aCompCor/aCompCor50 pipelines. Overall, in the (iii) stringent regime, the pipelines performed similarly one to another (with the sole exception of the 6HMP), and all pipelines performed better under the stringent regime than censoring‐based and lenient regimes, reducing significantly QC‐FC correlations. The inclusion of GSR barely changed any pipeline performance under this regime.

#### 3.2.2 QC‐FC distance‐dependence

As shown in Figure 3, the proportion of statistically significant correlations between QC‐FC and ROI distance for each denoising pipeline in each regime is comparable to prior validations (Parkes et al., 2018). Like QC‐FC, the stringent regime reduced distance‐dependence on QC‐FC the most (with an absolute average correlation of 0.06), followed by the lenient and the censoring‐based criteria (absolute average correlation of 0.16 and 0.25, respectively).

Specifically, in the (i) censoring‐based regime, pairing aCompCor with spike regression resulted in the lowest correlations (i.e., best performance) between distance and QC‐FC whether performed together with 6HMP, 24HMP, or GSR. Similarly, 24HMP with 2phys and spike regression also resulted in a low QF‐FC distance‐dependence. Like QC‐FC, the three worst pipelines all included data‐driven methods (ICA‐AROMA with 2phys; ICA‐AROMA with 2phys and GSR; and 24HMP with aCompCor50, and GSR). Likewise, scrubbing (with 24HMP, 2phys, with or without GSR) resulted in poor performance under this exclusion regime. Overall, the inclusion of GSR worsened pipeline performance across the board. Under the (ii) lenient regime, the combination of aCompCor with spike regression, whether with 6 or 24HMP, resulted in very low correlations (i.e., good performance), only surpassed by the combination of ICA‐AROMA with 2phys and 24HMP with 2phys and spike regression. The addition of GSR also worsened performance across all pipelines under this regime. aCompCor50 (with GSR) was the worst performer, followed by scrubbing paired with 24HMP, 2phys, and GSR, and 24HMP paired with 2phys and GSR. Finally, under the (iii) stringent regime, the combination of aCompCor and spike regression, whether with 6 or 24HMP, resulted in the lowest correlations (i.e., best performance). The addition of GSR generally resulted in unchanged or worse performance, with 24HMP with aCompCor50 and GSR resulting in the most significant absolute correlation.

#### 3.2.3 tDOF‐loss

**FIGURE 4:**
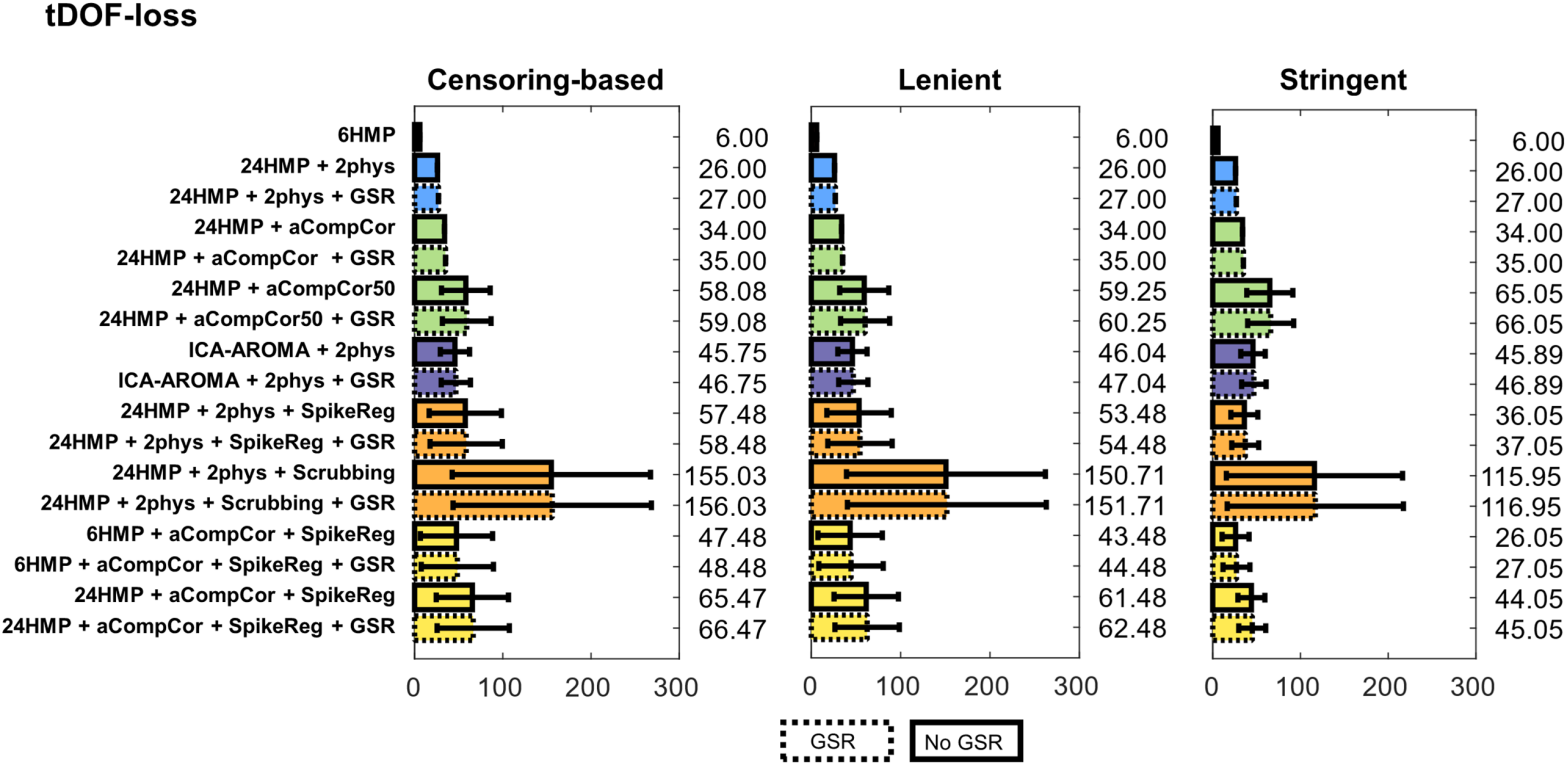
Temporal degrees of freedom loss (tDOF‐loss) under the three regimes of participant exclusion. Results are presented as mean ± standard deviation. Ideally, good denoising pipelines should use fewer regressors in the model, losing fewer degrees of freedom and resulting in values closer to 0. GSR, global signal regression.

As expected, the stringent regime resulted in the lowest average loss of tDOF (since the high‐movement subjects were excluded), albeit at the detriment of group degrees of freedom—given the large loss of sample size. Scrubbing resulted in the most significant tDOF‐loss across all regimes (Figure 4).

## 4 DISCUSSION

In this study, we report the first large‐scale evaluation of denoising pipelines in a clinical population known to be highly susceptible to in‐scanner motion (Hannawi et al., 2016) and to present considerable anatomical and functional abnormalities. Seventeen pipelines, obtained by combining nine denoising strategies, were evaluated for their ability to mitigate the effects of motion on rsfMRI connectivity estimates. Specifically, the pipelines were evaluated using three popular measures (Parkes et al., 2018): the reduction of correlations between functional connectivity and head motion (QC‐FC correlations), the reduction of the association between QC‐FC and distance between ROIs (QC‐FC distance‐dependence), and the loss of temporal degrees of freedom (tDOF‐loss). Overall, we report three main findings.

First, one of the most critical aspects of successful denoising is selecting which subjects to retain for further analysis (Satterthwaite et al., 2012, 2013; Van Dijk et al., 2012). In this high‐motion cohort, a stringent selection obviously resulted in equal or better performance in QC metrics across virtually all pipelines. In other words, once high‐motion participants are removed from the sample, the choice of denoising pipeline becomes secondary (with the sole exception of the 6HMP approach). Nonetheless, while the quality of the data used for analysis benefits significantly from this approach, it is very costly in terms of data loss (37% in our sample). Consequently, it decreases the degrees of freedom for statistical inference across groups (such as performing group comparison between patients and controls or correlation analysis between behavioral scores on a test of interest and FC metrics).

Second, different denoising approaches exhibit very distinct abilities to mitigate the negative effects of noise on FC (Parkes et al., 2018). Pipelines combining spike regression with 2phys and its extension, aCompCor, tend to be the best performers across exclusion regimes. Overall, pipelines using scrubbing were generally either comparable or worse than the other ones, in addition to the cost of two to three times greater loss of tDOF—up to 50% of the available data per subject—thus hampering the quality of the FC estimates. Pipelines containing data‐driven techniques (i.e., ICA‐AROMA and aCompCor50) were among the worst performers under most regimes. Such strategies are used in many studies since they retain the data's temporal structure (allowing frequency‐based analyses) and preserve the tDOF to a better extent than censoring strategies. The results involving ICA‐AROMA were somehow unexpected to us, as indeed ICA‐AROMA has been shown to perform very well in healthy volunteers or in patients with no evident brain damage (Parkes et al., 2018; Pruim, Mennes, Buitelaar, et al., 2015).

While it is hard to pinpoint the source of their poor performance in our analysis, we find that the segmentation of tissue compartments can be very problematic in the presence of significant brain shape deformation (e.g., due to primary impact damage, ventricular enlargement, and among others), which pose too great an obstacle to be addressed by denoising approaches that rely on proper brain segmentation to identify noise components. This is the case of ICA‐AROMA which uses, among the criteria to classify a component as noise, spatial features of each component, edge, and CSF fraction (Pruim, Mennes, van Rooij, et al., 2015), all of which rely on accurate structural preprocessing outcomes. TBI patients constitute a very heterogeneous sample from which segmenting the brain into different tissues might be challenging, probably affecting the performance of denoising strategies that depend on this step. In addition, ICA‐AROMA also uses spectral information to classify noise. Since motion (especially related to effects like spin‐history induced fluctuations) will show significant power at high frequencies, components will be classified as noise when they show the tendency toward increased power in the higher frequencies of the spectrum. However, patients with severe brain injury have altered spectral information. Indeed, ICA‐AROMA at times could not find any “signal” components in some patients (i.e., all components were classified as noise; Figure S1), stressing that these methods should be used with care when dealing with datasets containing pathological brains (Heine et al., 2012).

Third, we find the addition of GSR, a controversial step in fMRI data preprocessing, to give mixed results. GSR has been shown to reduce nonneuronal sources of physiological variance in the BOLD signal and to mitigate the effects of in‐scanner movement on FC metrics (Power et al., 2017; Yan et al., 2013). Nonetheless, removing the global signal inevitably eliminates the signal of interest (as the global signal is a superposition of both signal and noise components; Chen et al., 2012), and may introduce anticorrelations and alter the FC structure (Murphy et al., 2009; Weissenbacher et al., 2009). In our analysis, GSR improved the QC‐FC metric under the lenient and stringent regimes (albeit only very marginally in the latter), while it worsened the distance‐dependent QC metric for virtually all pipelines, under all regimes—consistent with prior reports (Ciric et al., 2017).

There are several limitations to the current work that should be acknowledged. First, our results are limited to the combination of approaches we chose for each pipeline. While some pipelines outperformed others, we should bear in mind that different combinations could yield divergent results (e.g., adding quadratic and derivative terms of physiological or global signal). Likewise, our results reflect the performance of pipelines for our particular image acquisition parameters. Testing these pipelines in images with shorter or longer TRs and other parameters should be addressed in future work. In addition, our analysis was performed under a project that enrolls moderate‐to‐severe TBI patients only and further studies should be conducted to evaluate whether our results endure in mild TBI. Second, the participants excluded from censoring pipelines (i.e., participants with <4 min of data based on spike regression or scrubbing) were also excluded from the other pipelines. While we thought it was crucial to compare pipelines maintaining the number of subjects constant across them, it also precluded us from evaluating how noncensoring pipelines would perform without this criterion. Future work should focus on assessing, for example, how data‐driven approaches perform when including these participants. Finally, our QC measures focused on a specific way of calculating FC (i.e., a model‐based method using Pearson's correlation between ROIs time series). We recognize that other metrics of FC (see Li et al., 2009) could result in different findings.

Many researchers have attempted to investigate denoising methods in a rigorous, systematic manner (Burgess et al., 2016; Ciric et al., 2018; Parkes et al., 2018; Pruim, Mennes, Buitelaar, et al., 2015; Raval et al., 2020), but defining the optimal approach is still a complex and open question. As rsfMRI has evolved to become an essential tool for examining brain networks in the healthy and diseased, it is critical to understand how best to model and account for artifact fluctuations. Overcoming noise‐related effects on rsfMRI can be even more challenging when researchers deal with high‐motion populations that present involuntary or abnormal movements, such as TBI patients (Hannawi et al., 2016). Furthermore, recent work has shown that rsfMRI can be used to supplement patient‐specific diagnosis and provide prognostic information from these patients who have no functional communication with their environment (Madhavan et al., 2019; Silva et al., 2015; Vanhaudenhuyse et al., 2010), highlighting the importance of rsfMRI not only in research but also in the clinical setting.

Finally, we offer three recommendations. First, where possible, use a stringent exclusion regime. That is, exclude any dataset for which mFD is greater than 0.25 mm, more than 20% of volumes present an FD greater than 0.2 mm, or any single volume presents FD greater than 5 mm (Satterthwaite et al., 2013). This approach essentially reduces the analyzed sample to low‐motion subjects, thus ensuring that systematic spurious correlations do not affect FC estimates. While the data loss can be sizeable (37% in our sample), this approach leads to the most significant mitigation of the negative effects of noise on FC. In addition, this strategy also gives the researcher freedom to choose among almost any pipeline, according to which procedure is best given the study's goals. However, this approach has the potential for biased data loss. For example, patients might be more motion‐prone, resulting in greater exclusion rates in a given clinical group and thus hampering group analyses (although there was no relationship between the level of consciousness measured by the Glasgow Coma Scale or any other demographic variable and motion in our sample). Second, when choosing between pipelines, we find combinations of 2phys, spike regression, and aCompCor to perform best in general. This combination outperforms, from our point of view, all other pipelines for reasons discussed above. Third, given the mixed results and the controversial nature of this step, our data argue against the use of GSR.

Taken together, our findings stress the heterogeneous performance of denoising pipelines, emphasizing that different strategies may be appropriate in the context of specific goals, according to the question, study design, and population investigated. Researchers should be familiar with their samples regarding head movement profile and clinical features and be aware of each approach's strengths and weaknesses to find the pipeline that best matches their goals. Findings such as these also highlight the crucial importance of large cross‐institution initiatives focused on best practices, rigor, and reproducibility (e.g., ENIGMA; Olsen et al., 2021) for functional MRI to be incorporated into routine clinical practice.

## AUTHOR CONTRIBUTIONS

The authors confirm contribution to the article as follows: Marina Weiler, Raphael F. Casseb, and Martin M. Monti conceived and designed the study; the Epilepsy Bioinformatics Study for Anti‐epileptogenic Therapy clinical trial (EpiBioS4Rx) research group (Table S4) collected the data; Marina Weiler, Raphael F. Casseb, Brunno M. de Campos, Julia S. Crone, Evan S. Lutkenhoff, Paul M. Vespa, and Martin M. Monti contributed with analysis tools; Marina Weiler and Raphael F. Casseb performed the analysis; Marina Weiler, Raphael F. Casseb, Brunno M. de Campos, Julia S. Crone, Evan S. Lutkenhoff, Paul M. Vespa, and Martin M. Monti wrote the article.

## CONFLICT OF INTEREST

The authors declare no conflicts of interest.

## Supporting information


**FIGURE S1:** Box plot showing the percentage of components classified as noise by ICA‐AROMA for each patient. When 100, it means that all components were classified as noise by the classifier.

**TABLE S1:** Detailed T1 image acquisition parameters.
**TABLE S2:** Detailed T2*‐weighted echo planar images acquisition parameters.
**TABLE S3:** Denoising pipelines and the total number of regressors used in each of them.
**TABLE S4:** EpiBioS4Rx's Principal Investigators and affiliated institutions.

## Data Availability

Anonymized data and code will be made available by request from any qualified investigator.
