# Supplementary figures and images for: Evaluating denoising strategies in resting‐state functional magnetic resonance in traumatic brain injury (EpiBioS4Rx)
Source: Hum Brain Mapp. 2022 Jun 20;43(15):4640–9. doi: 10.1002/hbm.25979 (PMC9491287; doi:10.1002/hbm.25979)

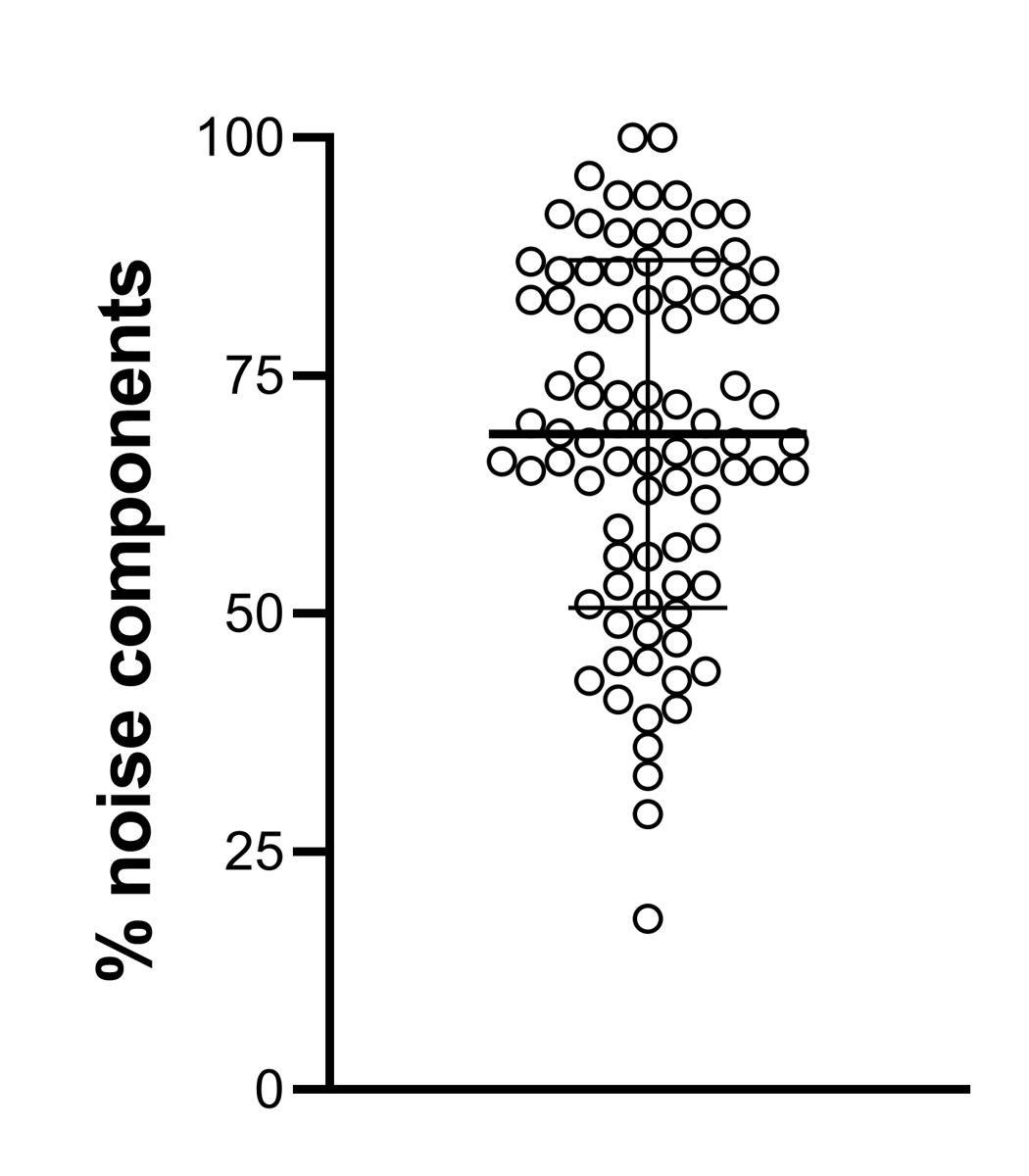

Supplement: Supplementary file 1 — FIGURE S1: Box plot showing the percentage of components classified as noise by ICA‐AROMA for each patient. When 100, it means that all components were classified as noise by the classifier. [file HBM-43-4640-s001.tif]
