# Supplementary material for: Evaluating denoising strategies in resting‐state functional magnetic resonance in traumatic brain injury (EpiBioS4Rx)
Source: Hum Brain Mapp. 2022 Jun 20;43(15):4640–9. doi: 10.1002/hbm.25979 (PMC9491287; doi:10.1002/hbm.25979)
Supplement: Supplementary file 2 — TABLE S1: Detailed T1 image acquisition parameters. TABLE S2: Detailed T2*‐weighted echo planar images acquisition parameters. TABLE S3: Denoising pipelines and the total number of regressors used in each of them. TABLE S4: EpiBioS4Rx's Principal Investigators and affiliated institutions. [file HBM-43-4640-s002.docx]

**Suppl. Table 1:** Detailed T1 image acquisition parameters:

| **Subj** | **Mag Field** | **Software** | **Model** | **Matrix** | | **Voxel Size** | | **Slice Thickness** | **Slices** | **TR (ms)** | **TE (ms)** | **Flip Angle** |
| --- | --- | --- | --- | --- | --- | --- | --- | --- | --- | --- | --- | --- |
| 1 | 3T | SIEMENS | Skyra | 256 | 256 | 1 | 1 | 1 | 256 | 2300 | 2.26 | 8 |
| 2 | 3T | SIEMENS | Skyra | 256 | 256 | 1 | 1 | 1 | 256 | 2300 | 2.26 | 8 |
| 3 | 3T | SIEMENS | Skyra | 256 | 256 | 1 | 1 | 1 | 256 | 2300 | 2.26 | 8 |
| 4 | 3T | SIEMENS | Skyra | 256 | 256 | 1 | 1 | 1 | 256 | 2300 | 2.26 | 8 |
| 5 | 3T | SIEMENS | Skyra | 256 | 256 | 1 | 1 | 1 | 256 | 2300 | 2.26 | 8 |
| 6 | 3T | SIEMENS | Skyra | 256 | 256 | 1 | 1 | 1 | 256 | 2300 | 2.26 | 8 |
| 7 | 3T | SIEMENS | Skyra | 256 | 256 | 1 | 1 | 1 | 256 | 2300 | 2.26 | 8 |
| 8 | 3T | SIEMENS | Skyra | 256 | 256 | 1 | 1 | 1 | 256 | 2300 | 2.26 | 8 |
| 9 | 3T | SIEMENS | Skyra | 256 | 256 | 1 | 1 | 1 | 256 | 2300 | 2.26 | 8 |
| 10 | 3T | SIEMENS | Skyra | 256 | 256 | 1 | 1 | 1 | 256 | 2300 | 2.26 | 8 |
| 11 | 3T | SIEMENS | Skyra | 256 | 256 | 1 | 1 | 1 | 256 | 2300 | 2.26 | 8 |
| 12 | 3T | Philips | Ingenia | 256 | 256 | 1 | 1 | 1 | 256 | 8.196 | 3.753 | 8 |
| 13 | 3T | Philips | Ingenia | 256 | 256 | 1 | 1 | 1 | 256 | 8.247 | 3.776 | 8 |
| 14 | 3T | Philips | Ingenia | 256 | 256 | 1 | 1 | 1 | 256 | 8.142 | 3.721 | 8 |
| 15 | 3T | Philips | Ingenia | 280 | 220 | 0.49 | 0.49 | 0.9 | 512 | 9.01 | 4.124 | 8 |
| 16 | 3T | Philips | Ingenia | 256 | 256 | 1 | 1 | 1 | 256 | 8.233 | 3.771 | 8 |
| 17 | 3T | Philips | Ingenia | 256 | 256 | 1 | 1 | 1 | 256 | 8.397 | 3.86 | 8 |
| 18 | 3T | GE | Signa | 256 | 256 | 1 | 1 | 1 | 256 | 8.856 | 3.488 | 15 |
| 19 | 3T | GE | Signa | 256 | 256 | 1 | 1 | 1 | 256 | 9.088 | 3.624 | 15 |
| 20 | 3T | GE | Signa | 256 | 256 | 1 | 1 | 1 | 256 | 8.824 | 3.488 | 15 |
| 21 | 3T | GE | Signa | 256 | 256 | 1 | 1 | 1 | 256 | 8.86 | 3.488 | 15 |
| 22 | 3T | GE | Signa | 256 | 256 | 1 | 1 | 1 | 256 | 8.868 | 3.488 | 15 |
| 23 | 3T | GE | Signa | 256 | 256 | 1 | 1 | 1 | 256 | 9.148 | 3.624 | 15 |
| 24 | 3T | GE | Signa | 256 | 256 | 1 | 1 | 1 | 256 | 11.28 | 4.848 | 20 |
| 25 | 3T | GE | Signa | 256 | 256 | 1 | 1 | 1 | 256 | 8.836 | 3.488 | 15 |
| 26 | 3T | GE | Signa | 256 | 256 | 1 | 1 | 1 | 256 | 9.116 | 3.524 | 15 |
| 27 | 3T | GE | Signa | 256 | 256 | 1 | 1 | 1 | 256 | 9.128 | 3.624 | 15 |
| 28 | 3T | GE | Signa | 256 | 256 | 1 | 1 | 1 | 256 | 9.136 | 3.624 | 15 |
| 29 | 3T | GE | Signa | 256 | 256 | 1 | 1 | 1 | 256 | 9.148 | 3.624 | 15 |
| 30 | 3T | GE | Signa | 256 | 256 | 1 | 1 | 1 | 256 | 9.076 | 3.624 | 15 |
| 31 | 3T | GE | Signa | 256 | 256 | 1 | 1 | 1 | 256 | 9.128 | 3.624 | 15 |
| 32 | 3T | GE | Signa | 256 | 256 | 1 | 1 | 1 | 256 | 9.148 | 3.616 | 15 |
| 33 | 3T | GE | Signa | 256 | 256 | 1 | 1 | 1 | 256 | 9.12 | 3.624 | 15 |
| 34 | 3T | GE | Signa | 256 | 256 | 1 | 1 | 1 | 256 | 9.096 | 3.624 | 15 |
| 35 | 3T | GE | Signa | 256 | 256 | 1 | 1 | 1 | 256 | 9.048 | 3.616 | 15 |
| 36 | 3T | GE | Signa | 256 | 256 | 1 | 1 | 1 | 256 | 9.096 | 3.616 | 15 |
| 37 | 3T | GE | Signa | 256 | 256 | 1 | 1 | 1 | 256 | 9.096 | 3.616 | 15 |
| 38 | 3T | GE | Signa | 256 | 256 | 1 | 1 | 1 | 256 | 9.148 | 3.616 | 15 |
| 39 | 3T | SIEMENS | TrioTim | 256 | 256 | 1 | 1 | 1 | 160 | 1900 | 3.43 | 9 |
| 40 | 3T | SIEMENS | TrioTim | 256 | 256 | 1 | 1 | 1 | 160 | 1900 | 3.43 | 9 |
| 41 | 3T | SIEMENS | TrioTim | 256 | 256 | 1 | 1 | 1 | 160 | 1900 | 3.43 | 9 |
| 42 | 3T | SIEMENS | TrioTim | 256 | 256 | 1 | 1 | 1 | 159 | 1900 | 3.43 | 9 |
| 43 | 3T | SIEMENS | TrioTim | 256 | 256 | 1 | 1 | 1 | 160 | 1900 | 3.43 | 9 |
| 44 | 3T | SIEMENS | TrioTim | 256 | 256 | 1 | 1 | 1 | 154 | 1900 | 3.43 | 9 |
| 45 | 3T | SIEMENS | TrioTim | 256 | 256 | 1 | 1 | 1 | 160 | 1900 | 3.43 | 9 |
| 46 | 3T | SIEMENS | TrioTim | 256 | 256 | 1 | 1 | 1 | 160 | 1900 | 3.43 | 9 |
| 47 | 3T | SIEMENS | TrioTim | 256 | 256 | 1 | 1 | 1 | 160 | 1900 | 3.43 | 9 |
| 48 | 3T | SIEMENS | TrioTim | 256 | 256 | 1 | 1 | 1 | 160 | 1900 | 3.43 | 9 |
| 49 | 3T | SIEMENS | TrioTim | 256 | 256 | 1 | 1 | 1 | 160 | 1900 | 3.43 | 9 |
| 50 | 3T | SIEMENS | TrioTim | 256 | 256 | 1 | 1 | 1 | 160 | 1900 | 3.43 | 9 |
| 51 | 3T | SIEMENS | TrioTim | 256 | 256 | 1 | 1 | 1 | 160 | 1900 | 3.43 | 9 |
| 52 | 3T | SIEMENS | TrioTim | 256 | 256 | 1 | 1 | 1 | 160 | 1900 | 3.43 | 9 |
| 53 | 3T | SIEMENS | TrioTim | 256 | 256 | 1 | 1 | 1 | 160 | 1900 | 3.43 | 9 |
| 54 | 3T | SIEMENS | TrioTim | 256 | 256 | 1 | 1 | 1 | 160 | 1900 | 3.43 | 9 |
| 55 | 3T | SIEMENS | TrioTim | 256 | 256 | 1 | 1 | 1 | 160 | 1900 | 3.43 | 9 |
| 56 | 3T | SIEMENS | TrioTim | 256 | 256 | 1 | 1 | 1 | 160 | 1900 | 3.43 | 9 |
| 57 | 3T | SIEMENS | TrioTim | 256 | 256 | 1 | 1 | 1 | 160 | 1900 | 3.43 | 9 |
| 58 | 3T | SIEMENS | TrioTim | 256 | 256 | 1 | 1 | 1 | 160 | 1900 | 3.43 | 9 |
| 59 | 3T | SIEMENS | TrioTim | 256 | 256 | 1 | 1 | 1 | 160 | 1900 | 3.43 | 9 |
| 60 | 3T | SIEMENS | TrioTim | 256 | 256 | 1 | 1 | 1 | 160 | 1900 | 3.43 | 9 |
| 61 | 1.5T | SIEMENS | Aera | 256 | 256 | 1 | 1 | 1 | 160 | 2000 | 3.13 | 15 |
| 62 | 1.5T | SIEMENS | Aera | 256 | 256 | 1 | 1 | 1 | 192 | 2000 | 3.13 | 15 |
| 63 | 1.5T | SIEMENS | Aera | 256 | 256 | 1 | 1 | 1 | 160 | 2000 | 3.13 | 15 |
| 64 | 1.5T | SIEMENS | Aera | 256 | 256 | 1 | 1 | 1 | 144 | 2000 | 3.13 | 15 |
| 65 | 1.5T | SIEMENS | Aera | 256 | 256 | 1 | 1 | 1 | 160 | 2000 | 3.13 | 15 |
| 66 | 1.5T | SIEMENS | Aera | 256 | 256 | 1 | 1 | 1 | 128 | 2000 | 3.13 | 15 |
| 67 | 1.5T | SIEMENS | Aera | 256 | 256 | 1 | 1 | 1 | 144 | 2000 | 3.13 | 15 |
| 68 | 1.5T | SIEMENS | Aera | 256 | 256 | 1 | 1 | 1 | 176 | 2000 | 3.13 | 15 |
| 69 | 1.5T | SIEMENS | Aera | 256 | 256 | 1 | 1 | 1 | 176 | 2000 | 3.13 | 15 |
| 70 | 1.5T | SIEMENS | Aera | 256 | 256 | 1 | 1 | 1 | 128 | 2000 | 3.13 | 15 |
| 71 | 1.5T | SIEMENS | Aera | 256 | 256 | 1 | 1 | 1 | 160 | 2000 | 3.13 | 15 |
| 72 | 1.5T | SIEMENS | Aera | 256 | 256 | 1 | 1 | 1 | 176 | 2000 | 3.13 | 15 |
| 73 | 1.5T | SIEMENS | Aera | 256 | 256 | 1 | 1 | 1 | 128 | 2000 | 3.13 | 15 |
| 74 | 1.5T | SIEMENS | Aera | 256 | 256 | 1 | 1 | 1 | 192 | 2000 | 3.13 | 15 |
| 75 | 1.5T | SIEMENS | Aera | 256 | 256 | 1 | 1 | 1 | 144 | 2000 | 3.13 | 15 |
| 76 | 1.5T | SIEMENS | Aera | 256 | 256 | 1 | 1 | 1 | 144 | 2000 | 3.13 | 15 |
| 77 | 1.5T | SIEMENS | Aera | 256 | 256 | 1 | 1 | 1 | 144 | 2000 | 3.13 | 15 |
| 78 | 3T | SIEMENS | Verio | 256 | 256 | 1 | 1 | 1 | 160 | 1900 | 2.93 | 9 |
| 79 | 3T | SIEMENS | Verio | 256 | 256 | 1 | 1 | 1 | 160 | 1900 | 2.93 | 9 |
| 80 | 3T | SIEMENS | Verio | 256 | 256 | 1 | 1 | 1 | 176 | 1900 | 2.93 | 9 |
| 81 | 3T | SIEMENS | Verio | 256 | 256 | 1 | 1 | 1 | 160 | 1900 | 2.93 | 9 |
| 82 | 3T | SIEMENS | Verio | 256 | 256 | 1 | 1 | 1 | 176 | 1900 | 2.93 | 9 |
| 83 | 3T | SIEMENS | Skyra | 256 | 256 | 1 | 1 | 1 | 256 | 2300 | 2.26 | 8 |
| 84 | 3T | SIEMENS | Skyra | 256 | 256 | 1 | 1 | 1 | 256 | 2300 | 2.26 | 8 |
| 85 | 3T | SIEMENS | Skyra | 256 | 256 | 1 | 1 | 1 | 256 | 2300 | 2.26 | 8 |
| 86 | 3T | SIEMENS | Skyra | 256 | 256 | 1 | 1 | 1 | 256 | 2300 | 2.26 | 8 |
| 87 | 3T | SIEMENS | Skyra | 256 | 256 | 1 | 1 | 1 | 256 | 2300 | 2.26 | 8 |
| 88 | 3T | SIEMENS | Skyra | 256 | 256 | 1 | 1 | 1 | 256 | 2300 | 2.26 | 8 |

**Suppl. Table 2:** Detailed T2*-weighted echo planar images acquisition parameters:

| **Subj** | **Mag Field** | **Software** | **Model** | **Matrix** | | **Voxel Size** | | **Slice Thickness** | **Slices** | **TR (ms)** | **TE (ms)** | **Flip Angle** | **Volumes** |
| --- | --- | --- | --- | --- | --- | --- | --- | --- | --- | --- | --- | --- | --- |
| 1 | 3T | SIEMENS | Skyra | 64 | 64 | 3.4375 | 3.4375 | 4.25 | 37 | 2000 | 25.000 | 78 | 300 |
| 2 | 3T | SIEMENS | Skyra | 64 | 64 | 3.4375 | 3.4375 | 4.25 | 37 | 2000 | 25.000 | 78 | 300 |
| 3 | 3T | SIEMENS | Skyra | 64 | 64 | 3.4375 | 3.4375 | 4.25 | 37 | 2000 | 25.000 | 78 | 300 |
| 4 | 3T | SIEMENS | Skyra | 64 | 64 | 3.4375 | 3.4375 | 4.25 | 37 | 2000 | 25.000 | 78 | 300 |
| 5 | 3T | SIEMENS | Skyra | 64 | 64 | 3.4375 | 3.4375 | 4.25 | 38 | 2000 | 25.000 | 78 | 300 |
| 6 | 3T | SIEMENS | Skyra | 64 | 64 | 3.4375 | 3.4375 | 4.25 | 37 | 2000 | 25.000 | 78 | 300 |
| 7 | 3T | SIEMENS | Skyra | 64 | 64 | 3.4375 | 3.4375 | 4.25 | 37 | 2000 | 25.000 | 78 | 300 |
| 8 | 3T | SIEMENS | Skyra | 64 | 64 | 3.4375 | 3.4375 | 4.25 | 37 | 2000 | 25.000 | 78 | 300 |
| 9 | 3T | SIEMENS | Skyra | 64 | 64 | 3.9063 | 3.9063 | 4.25 | 42 | 2290 | 25.000 | 78 | 300 |
| 10 | 3T | SIEMENS | Skyra | 64 | 64 | 3.4375 | 3.4375 | 4.25 | 37 | 2000 | 25.000 | 78 | 300 |
| 11 | 3T | SIEMENS | Skyra | 64 | 64 | 3.4375 | 3.4375 | 4.25 | 37 | 2000 | 25.000 | 78 | 300 |
| 12 | 3T | Philips | Ingenia | 80 | 80 | 2.75 | 2.75 | 3.65 | 37 | 2000 | 25.001 | 78 | 300 |
| 13 | 3T | Philips | Ingenia | 80 | 80 | 2.75 | 2.75 | 3.65 | 37 | 2000 | 25.001 | 78 | 300 |
| 14 | 3T | Philips | Ingenia | 80 | 80 | 2.75 | 2.75 | 3.65 | 37 | 2000 | 25.001 | 78 | 300 |
| 15 | 3T | Philips | Ingenia | 80 | 80 | 2.75 | 2.75 | 3.65 | 37 | 2000 | 25.001 | 78 | 300 |
| 16 | 3T | Philips | Ingenia | 80 | 80 | 2.75 | 2.75 | 3.65 | 37 | 2000 | 25.001 | 78 | 300 |
| 17 | 3T | Philips | Ingenia | 80 | 80 | 2.75 | 2.75 | 3.65 | 37 | 2000 | 25.001 | 78 | 300 |
| 18 | 3T | GE | Signa | 64 | 64 | 3.75 | 3.75 | 3.70 | 40 | 2000 | 25.000 | 90 | 300 |
| 19 | 3T | GE | Signa | 64 | 64 | 3.4375 | 3.4375 | 3.70 | 40 | 2000 | 25.000 | 90 | 300 |
| 20 | 3T | GE | Signa | 64 | 64 | 3.4375 | 3.4375 | 3.70 | 40 | 2000 | 25.000 | 90 | 300 |
| 21 | 3T | GE | Signa | 64 | 64 | 3.75 | 3.75 | 3.70 | 40 | 2100 | 25.000 | 90 | 300 |
| 22 | 3T | GE | Signa | 64 | 64 | 3.4375 | 3.4375 | 3.70 | 40 | 2000 | 25.000 | 90 | 300 |
| 23 | 3T | GE | Signa | 64 | 64 | 3.75 | 3.75 | 3.70 | 40 | 2000 | 25.000 | 90 | 300 |
| 24 | 3T | GE | Signa | 64 | 64 | 3.4375 | 3.4375 | 3.70 | 38 | 2000 | 25.000 | 78 | 300 |
| 25 | 3T | GE | Signa | 64 | 64 | 3.4375 | 3.4375 | 3.70 | 44 | 2200 | 25.000 | 78 | 300 |
| 26 | 3T | GE | Signa | 64 | 64 | 3.4375 | 3.4375 | 3.70 | 46 | 2300 | 25.000 | 78 | 300 |
| 27 | 3T | GE | Signa | 64 | 64 | 3.4375 | 3.4375 | 3.70 | 39 | 2000 | 25.000 | 78 | 300 |
| 28 | 3T | GE | Signa | 64 | 64 | 3.4375 | 3.4375 | 3.70 | 40 | 2000 | 25.000 | 78 | 300 |
| 29 | 3T | GE | Signa | 64 | 64 | 3.4375 | 3.4375 | 3.70 | 42 | 2100 | 25.000 | 78 | 300 |
| 30 | 3T | GE | Signa | 64 | 64 | 3.4375 | 3.4375 | 3.70 | 42 | 2138 | 25.000 | 78 | 300 |
| 31 | 3T | GE | Signa | 64 | 64 | 3.4375 | 3.4375 | 3.70 | 45 | 2297 | 25.000 | 78 | 300 |
| 32 | 3T | GE | Signa | 64 | 64 | 3.4375 | 3.4375 | 3.70 | 40 | 2000 | 25.000 | 78 | 300 |
| 33 | 3T | GE | Signa | 64 | 64 | 3.4375 | 3.4375 | 3.70 | 43 | 2150 | 25.000 | 78 | 300 |
| 34 | 3T | GE | Signa | 64 | 64 | 3.4375 | 3.4375 | 3.70 | 40 | 2000 | 25.000 | 78 | 300 |
| 35 | 3T | GE | Signa | 64 | 64 | 3.4375 | 3.4375 | 3.70 | 40 | 2113 | 25.000 | 78 | 300 |
| 36 | 3T | GE | Signa | 64 | 64 | 3.4375 | 3.4375 | 3.70 | 40 | 2000 | 25.000 | 78 | 300 |
| 37 | 3T | GE | Signa | 64 | 64 | 3.4375 | 3.4375 | 3.70 | 40 | 2000 | 25.000 | 78 | 300 |
| 38 | 3T | GE | Signa | 64 | 64 | 3.4375 | 3.4375 | 3.70 | 44 | 2200 | 25.000 | 78 | 300 |
| 39 | 3T | SIEMENS | TrioTim | 64 | 64 | 3.4375 | 3.4375 | 4.25 | 37 | 2000 | 25.000 | 90 | 300 |
| 40 | 3T | SIEMENS | TrioTim | 64 | 64 | 3.4375 | 3.4375 | 4.25 | 37 | 2000 | 25.000 | 90 | 300 |
| 41 | 3T | SIEMENS | TrioTim | 64 | 64 | 3.4375 | 3.4375 | 4.25 | 37 | 2000 | 25.000 | 90 | 300 |
| 42 | 3T | SIEMENS | TrioTim | 64 | 64 | 3.4375 | 3.4375 | 4.25 | 37 | 2000 | 25.000 | 90 | 300 |
| 43 | 3T | SIEMENS | TrioTim | 64 | 64 | 3.4375 | 3.4375 | 4.25 | 37 | 2000 | 25.000 | 90 | 300 |
| 44 | 3T | SIEMENS | TrioTim | 64 | 64 | 3.4375 | 3.4375 | 4.25 | 37 | 2000 | 25.000 | 90 | 300 |
| 45 | 3T | SIEMENS | TrioTim | 64 | 64 | 3.4375 | 3.4375 | 4.25 | 37 | 2000 | 25.000 | 90 | 300 |
| 46 | 3T | SIEMENS | TrioTim | 64 | 64 | 3.4375 | 3.4375 | 4.25 | 37 | 2000 | 25.000 | 90 | 300 |
| 47 | 3T | SIEMENS | TrioTim | 64 | 64 | 3.4375 | 3.4375 | 4.25 | 37 | 2000 | 25.000 | 90 | 300 |
| 48 | 3T | SIEMENS | TrioTim | 64 | 64 | 3.4375 | 3.4375 | 4.25 | 37 | 2000 | 25.000 | 90 | 300 |
| 49 | 3T | SIEMENS | TrioTim | 64 | 64 | 3.4375 | 3.4375 | 4.25 | 37 | 2000 | 25.000 | 90 | 300 |
| 50 | 3T | SIEMENS | TrioTim | 64 | 64 | 3.4375 | 3.4375 | 4.25 | 37 | 2000 | 25.000 | 90 | 300 |
| 51 | 3T | SIEMENS | TrioTim | 64 | 64 | 3.4375 | 3.4375 | 4.25 | 37 | 2000 | 25.000 | 90 | 300 |
| 52 | 3T | SIEMENS | TrioTim | 64 | 64 | 3.4375 | 3.4375 | 4.25 | 37 | 2000 | 25.000 | 78 | 300 |
| 53 | 3T | SIEMENS | TrioTim | 64 | 64 | 3.4375 | 3.4375 | 4.25 | 37 | 2000 | 25.000 | 78 | 300 |
| 54 | 3T | SIEMENS | TrioTim | 64 | 64 | 3.4375 | 3.4375 | 4.25 | 37 | 2000 | 25.000 | 78 | 300 |
| 55 | 3T | SIEMENS | TrioTim | 64 | 64 | 3.4375 | 3.4375 | 4.25 | 37 | 2000 | 25.000 | 78 | 300 |
| 56 | 3T | SIEMENS | TrioTim | 64 | 64 | 3.4375 | 3.4375 | 4.25 | 37 | 2000 | 25.000 | 78 | 300 |
| 57 | 3T | SIEMENS | TrioTim | 64 | 64 | 3.4375 | 3.4375 | 4.25 | 37 | 2000 | 25.000 | 78 | 300 |
| 58 | 3T | SIEMENS | TrioTim | 64 | 64 | 3.4375 | 3.4375 | 4.25 | 37 | 2000 | 25.000 | 78 | 300 |
| 59 | 3T | SIEMENS | TrioTim | 64 | 64 | 3.4375 | 3.4375 | 4.25 | 37 | 2000 | 25.000 | 78 | 300 |
| 60 | 3T | SIEMENS | TrioTim | 64 | 64 | 3.4375 | 3.4375 | 4.25 | 37 | 2000 | 25.000 | 78 | 300 |
| 61 | 1.5T | SIEMENS | Aera | 64 | 64 | 3.4375 | 3.4375 | 4.48 | 34 | 2000 | 25.000 | 78 | 300 |
| 62 | 1.5T | SIEMENS | Aera | 64 | 64 | 3.4375 | 3.4375 | 4.48 | 34 | 2000 | 25.000 | 78 | 300 |
| 63 | 1.5T | SIEMENS | Aera | 64 | 64 | 3.75 | 3.75 | 4.48 | 34 | 2000 | 25.000 | 78 | 300 |
| 64 | 1.5T | SIEMENS | Aera | 64 | 64 | 3.4375 | 3.4375 | 4.48 | 34 | 2070 | 25.000 | 78 | 300 |
| 65 | 1.5T | SIEMENS | Aera | 64 | 64 | 3.4375 | 3.4375 | 3.81 | 41 | 2500 | 25.000 | 78 | 300 |
| 66 | 1.5T | SIEMENS | Aera | 64 | 64 | 3.4375 | 3.4375 | 3.81 | 34 | 2000 | 25.000 | 78 | 300 |
| 67 | 1.5T | SIEMENS | Aera | 64 | 64 | 3.4375 | 3.4375 | 3.81 | 34 | 2000 | 25.000 | 78 | 300 |
| 68 | 1.5T | SIEMENS | Aera | 64 | 64 | 3.4375 | 3.4375 | 3.81 | 34 | 2000 | 25.000 | 78 | 300 |
| 69 | 1.5T | SIEMENS | Aera | 64 | 64 | 3.4375 | 3.4375 | 3.81 | 41 | 2370 | 25.000 | 78 | 300 |
| 70 | 1.5T | SIEMENS | Aera | 64 | 64 | 3.4375 | 3.4375 | 3.81 | 34 | 2000 | 25.000 | 78 | 300 |
| 71 | 1.5T | SIEMENS | Aera | 64 | 64 | 3.4375 | 3.4375 | 3.81 | 34 | 2000 | 25.000 | 78 | 300 |
| 72 | 1.5T | SIEMENS | Aera | 64 | 64 | 3.5938 | 3.5938 | 3.81 | 38 | 2200 | 25.000 | 78 | 300 |
| 73 | 1.5T | SIEMENS | Aera | 64 | 64 | 3.4375 | 3.4375 | 3.81 | 34 | 2000 | 25.000 | 78 | 300 |
| 74 | 1.5T | SIEMENS | Aera | 64 | 64 | 3.4375 | 3.4375 | 3.81 | 45 | 2600 | 25.000 | 78 | 300 |
| 75 | 1.5T | SIEMENS | Aera | 64 | 64 | 3.4375 | 3.4375 | 3.81 | 37 | 2140 | 25.000 | 78 | 300 |
| 76 | 1.5T | SIEMENS | Aera | 64 | 64 | 3.4375 | 3.4375 | 3.81 | 34 | 2000 | 25.000 | 78 | 300 |
| 77 | 1.5T | SIEMENS | Aera | 64 | 64 | 3.4375 | 3.4375 | 3.81 | 34 | 2000 | 25.000 | 78 | 300 |
| 78 | 3T | SIEMENS | Verio | 64 | 64 | 3.4375 | 3.4375 | 3.91 | 36 | 2000 | 25.000 | 78 | 300 |
| 79 | 3T | SIEMENS | Verio | 64 | 64 | 3.4375 | 3.4375 | 3.91 | 34 | 2000 | 25.000 | 78 | 300 |
| 80 | 3T | SIEMENS | Verio | 64 | 64 | 3.4375 | 3.4375 | 3.91 | 34 | 2000 | 25.000 | 78 | 300 |
| 81 | 3T | SIEMENS | Verio | 64 | 64 | 3.4375 | 3.4375 | 3.91 | 34 | 2000 | 25.000 | 78 | 300 |
| 82 | 3T | SIEMENS | Verio | 64 | 64 | 3.4375 | 3.4375 | 3.91 | 40 | 2000 | 25.000 | 78 | 300 |
| 83 | 3T | SIEMENS | Skyra | 64 | 64 | 3.4375 | 3.4375 | 4.25 | 37 | 2000 | 25.000 | 78 | 300 |
| 84 | 3T | SIEMENS | Skyra | 64 | 64 | 3.4375 | 3.4375 | 4.25 | 37 | 2000 | 25.000 | 78 | 300 |
| 85 | 3T | SIEMENS | Skyra | 64 | 64 | 3.4375 | 3.4375 | 4.25 | 37 | 2000 | 25.000 | 78 | 300 |
| 86 | 3T | SIEMENS | Skyra | 64 | 64 | 3.4375 | 3.4375 | 4.25 | 37 | 2000 | 25.000 | 78 | 300 |
| 87 | 3T | SIEMENS | Skyra | 64 | 64 | 3.4375 | 3.4375 | 4.25 | 37 | 2000 | 25.000 | 78 | 300 |
| 88 | 3T | SIEMENS | Skyra | 64 | 64 | 3.4375 | 3.4375 | 4.25 | 37 | 2000 | 25.000 | 78 | 300 |

**Suppl. Table 3:** Denoising pipelines and the total number of regressors used in each of them.

| **Denoising Pipeline** | **nr. of regressors** |
| --- | --- |
| 6HMP | 6 |
| 24HMP + 2Phys | 26 |
| 24HMP + 2Phys + GSR | 27 |
| 24HMP + aCompCor | 34 |
| 24HMP + aCompCor + GSR | 35 |
| 24HMP + aCompCor50 | 24 + *k* |
| 24HMP + aCompCor50 + GSR | 25 + *k* |
| 2Phys + ICA-AROMA | 2 + *k* |
| 2Phys + ICA-AROMA + GSR | 3 + *k* |
| 24HMP + 2Phys + SpikeReg | 26 + *k* |
| 24HMP + 2Phys + SpikeReg + GSR | 27 + *k* |
| 24HMP + 2Phys + Scrubbing | 26 + *k* |
| 24HMP + 2Phys + Scrubbing + GSR | 27 + *k* |
| 6HMP + aCompCor + SpikeReg | 16 + *k* |
| 6HMP + aCompCor + SpikeReg + GSR | 17 + *k* |
| 24HMP + aCompCor + SpikeReg | 34 + *k* |
| 24HMP + aCompCor +SpikeReg + GSR | 35 + *k* |

Abbreviations: HMP = Head Motion Parameters; Phys = physiological regressors (white matter (WM) and cerebrospinal fluid (CSF) average time-series); GSR = Global Signal Regression; aCompCor = anatomical Component Correction; SpikeReg = Spike regression.

*k* = variable that represents the number of additional regressors estimated by the denoising method, which varies from subject to subject. For aCompCor50, *k* is the number of components that explain 50% of the variance in each WM and CSF compartments; for ICA-AROMA, *k* represents the total number of components classified as noise; for SpikeReg and Scrubbing, *k* represents the number of contaminated volumes. Ideally, good denoising pipelines should use fewer regressors in the model, losing fewer degrees of freedom.

**Suppl. Table 4**: EpiBioS4Rx’s Principal Investigators and affiliated institutions.

| **Principal Investigator/Author** | **Affiliated Institutions** |
| --- | --- |
| Alaa Kamnaksh | Uniformed Services University, United States |
| Alicia Au | University of Pittsburgh, United States |
| Andrew Morokoff | Royal Melbourne Hospital, United States |
| Arthur Toga | University of Southern California, United States |
| Ben Ellingson | David Geffen School of Medicine at UCLA, United States |
| Brandon Foreman | University of Cincinnati, United States |
| Courtney L. Robertson | Johns Hopkins University, United States |
| Courtney Real | David Geffen School of Medicine at UCLA, United States |
| David McArthur | David Geffen School of Medicine at UCLA, United States |
| Denes Agoston | Uniformed Services University, United States |
| Elisa Yam | David Geffen School of Medicine at UCLA, United States |
| Emily J. Gilmore | Yale University, United States |
| Eric Rosenthal | Harvard University/Massachusetts General Hospital, United States |
| Jan Claassen | Columbia University, United States |
| Lara Zimmermann | University of California, Davis, United States |
| Lawrence Hirsch | Yale University, United States |
| Manuel Buitrago Blanco | David Geffen School of Medicine at UCLA, United States |
| Michael J. Bell | Children's National Health System, United States |
| Ramon Diaz-Arrastia | University of Pennsylvania, United States |
| Richard Staba | David Geffen School of Medicine at UCLA, United States |
| Susana Martinez | David Geffen School of Medicine at UCLA, United States |
| Terrence O'Brien | Royal Melbourne Hospital, United States |
| Dominique Duncan | University of Southern California, United States |
| Frederick A. Willyerd | Phoenix Children's Hospital, United States |
| Jerome Engel Jr. | David Geffen School of Medicine at UCLA, United States |
| Kristine O'Phelan | University of Miami, United States |
| Martin Hunn | The Alfred Hospital Melbourne, Australia |
| Nicholas Abend | University of Pennsylvania, United States |
| Thomas P. Bleck | Rush University, United States |
| Vikesh Shrestha | David Geffen School of Medicine at UCLA, United States |
